# Hydrogen production by the steam reforming of synthetic biogas in atmospheric-pressure microwave (915 MHz) plasma

**DOI:** 10.1038/s41598-023-29433-y

**Published:** 2023-02-07

**Authors:** Bartosz Hrycak, J. Mizeraczyk, D. Czylkowski, M. Dors, M. Budnarowska, M. Jasiński

**Affiliations:** 1 Institute of Fluid Flow Machinery, Polish Academy of Sciences, Fiszera 14, 80-231 Gdańsk, Poland; 2 Department of Marine Electronics, Gdynia Maritime University, Morska 81-87, 81-225 Gdynia, Poland

**Keywords:** Chemical engineering, Plasma physics

## Abstract

This paper is a contribution to the development of microwave plasma-based technology aimed at efficient hydrogen (H_2_) production from a so-called synthetic biogas, considered a mixture of methane (CH_4_) and carbon dioxide (CO_2_), which can contain up to 70% CH_4_. In this work, we tested the performance of a waveguide-supplied metal cylinder-based microwave plasma source (MPS) operating at 915 MHz at atmospheric pressure as a tool for the efficient production of H_2_ in the steam reforming of the synthetic biogas. The test showed that the steam reforming of the synthetic biogas could be carried out under a wide range of working parameters without soot formation and extinction of the microwave discharge. We found that there is a minimal H_2_O_steam_ consumption rate for a given CH_4_ input volume content, which ensures stable operation of the MPS (no soot). The experiments did not show that increasing the amount of H_2_O_steam_ rate above the minimal value for a given CH_4_ input volume content results in an increase in the H_2_ production rate, energy yield, CH_4_ conversion degree, and H_2_ output concentration. To describe the MPS performance, which also takes into account a factor of the utilization of the CH_4_ feedstock, we introduced a new parameter, called an energy–CH_4_ feedstock consumption yield. The best results in terms of the H_2_ production rate, the energy yield, and the CH_4_ conversion degree were 239 g[H_2_]/h 36.8 g[H_2_]/kWh, and 74.3%, respectively. This shows that the application of the steam reforming, instead of the dry reforming, resulted in a 1.5-fold increase of the H_2_ production rate and the corresponding energy yield.

## Introduction

The thread of global warming and the declining resources of fossil fuels force the newly developed energy source to meet the requirements of renewable energy and environmental friendliness at the same time. One of the most promising renewable and ecologically clean energy sources seems to be hydrogen (H_2_). Many reviews described technologies related to the production of H_2_ from various resources^1–4^.

In the future hydrogen is predicted to be used not as a fuel to produce heat by combustion^5^ but rather as an energy carrier to activate fuel cells^6–9^. This increases the interest in new sources and methods of hydrogen production. As an alternative to methane (CH_4_), which so far has been a common source of hydrogen^2^, biogas is considered to be renewable and ecological.

A wide variety of raw materials such as green waste, agricultural waste, municipal waste, household waste, sewage etc. can be used to produce biogas^10^. The components of biogas from landfills, waste water treatment plants (WWTP), sludge digesters and biogas plants processing various materials are methane (CH_4_), carbon dioxide (CO_2_), oxygen (O_2_), nitrogen (N_2_), ammonia (NH_3_), volatile organic compounds (VOCs) including organic silicon compounds, halogenated compounds and sulphur compounds (mainly hydrogen sulphide (H_2_S))^11,12^. CH_4_ and CO_2_ are the main compounds of a typical biogas. The content of CH_4_ and CO_2_ in biogas from landfills, WWTPs and biogas plants usually ranges from 50 to 70% and from 35 to 45%, respectively. Biogas contains also: N_2_ (1–3%) and O_2_ (less than 1%). Other biogas components can be: water vapour (H_2_O), carbon monoxide (CO) and solid particles.

The CO_2_ contained in biogas produced from plants is absorbed by plants from the atmosphere. Therefore its origin is ecologically neutral. The continuous production-and-use cycle of biogas does not generate any net CO_2_. This cause that biogas can be consider as renewable and ecological H_2_ source^11,13^.

Many applications require that some components must be removed from biogas and a higher concentration of CH_4_ in the biogas have to be ensured^1,14^. For example, the typical concentration of corrosive H_2_S in the raw biogas is sufficient to destroy biogas installations and devices^15^. In order to have the same standards as fossil natural gas, the biogas needs to be cleaned and the concentration of CH_4_ needs to be raised up to natural gas standards to become bio-methane (98% CH_4_ content). Such an upgraded biogas is capable of being used in local natural gas networks. In particular, both cleaning and upgrading are most likely required if high purity H_2_ for fuel cell activation is expected to be obtained from the biogas.

Several conventional CH_4_ reforming processes (pyrolysis, steam reforming, dry reforming, partial oxidation, auto-thermal reforming) can be adopted to produce H_2_ from biogas, since the major component of biogas is CH_4_:

1 $${\text{CH}}_{{4}} \to {\text{C }} + {\text{ 2H}}_{{2}} \left( {{\text{pyrolysis}}} \right),$$

2 $${\text{CH}}_{{4}} + {\text{ H}}_{{2}} {\text{O}} \to {\text{CO }} + {\text{ 3H}}_{{2}} \left( {\text{steam reforming}} \right),$$

3 $${\text{CH}}_{{4}} + {\text{ CO}}_{{2}} \to {\text{2CO }} + {\text{ 2H}}_{{2}} \left( {\text{dry reforming}} \right),$$

4 $${\text{CH}}_{{4}} + \, 0.{\text{5O}}_{{2}} \to {\text{CO }} + {\text{ 2H}}_{{2}} \left( {\text{partial oxidation}} \right),$$

5a $${\text{2CH}}_{{4}} + {\text{ O}}_{{2}} + {\text{ CO}}_{{2}} \to {\text{3CO }} + {\text{ 3H}}_{{2}} + {\text{ H}}_{{2}} {\text{O }}\left( {{\text{auto}}-{\text{thermal reforming}}} \right),$$

5b $${\text{4CH}}_{{4}} + {\text{ O}}_{{2}} + {\text{ 2H}}_{{2}} {\text{O}} \to {\text{4CO }} + { 1}0{\text{H}}_{{2}} \left( {{\text{auto}}-{\text{thermal reforming}}} \right).$$

All conventional methods (1)–(5a, 5b) of H_2_ production have been widely discussed in many publications^11–13,16^. Some of these conventional reforming technologies mentioned above are already or will be soon commercialized (steam reforming, partial oxidation reforming and auto-thermal reforming).

Over the last two decades an alternative technology to produce H_2_ has been proposed and developed. This technology is based on thermal and non-thermal plasmas generated by electrical discharges. Such plasmas are used to reform gaseous and liquid hydrogen carriers to produce H_2_^17–29^. These discharges are: glow discharges^30^, corona discharges^31,32^, arc discharges^33–36^, dielectric barrier discharges (DBD)^15,37–39^ and microwave discharges^40–44^.

The microwave plasma is an ionized gas sustained by an electromagnetic field of high frequency (0.3–300 GHz). A technologically important advantage of a microwave discharge over other discharges is the lack of internal electrodes, which require regular maintenance or/and replacement due to erosion. Microwave discharges can be generated over a wide pressure range (from a mbar to a few bar) of the working gas. Moreover, the efficiency of energy transfer from the electromagnetic field to the plasma is very high (up to 90%). Microwave plasma is characterized by a relatively high concentration and energy of electrons. This cause a high concentration of active ingredients that enhance the chemical reactions in the working gas. The microwave discharges generate one of the most promising plasma environments for the production of H_2_ from CH_4_-containing gases, in particular biogas.

Our research to date showed that microwave plasma-based technologies at 2.45 GHz for the steam reforming of synthetic biogas^42^ and at 915 MHz used for the dry reforming of synthetic biogas^44^ revealed the limitations of both microwave plasma technologies in terms of the H_2_ production efficiency, determined by the H_2_ production rate and energy yield. Firstly, the use of classical dry reforming of synthetic biogas in a 915 MHz microwave system resulted in a relatively high (156 [g(H_2_)/h]) rate of hydrogen production and energy yield (about 21 g(H_2_)/kWh)^44^ compared to other plasma methods. However, a further increase of these parameters was inhibited by soot formation on the quartz tube inner surface and in the plasma zone which led to choking and extinguishing the microwave discharge when the CH_4_ input volume content increased above 40%. This suggests that the limitation of the CH_4_ input volume content to 40% in the microwave plasma dry reforming of biogas in a 915 MHz microwave system is the major obstacle to approaching the energy yield of H_2_ production of 60 g(H_2_)/kWh, which is recognized by the U.S. Department of Energy (DOE) as an adopted target for small-scale technologies intended for industrial distributed hydrogen production^5^. Following the conclusions of our previous investigation in the steam reforming of biogas-like mixtures in a 2.45 GHz microwave system^42^, this steam reforming could be an effective way to overcome the CH_4_ input volume content limitation encountered in 915 MHz microwave dry plasma reforming. Secondly, our previous research in the steam reforming of biogas-like mixtures in a 2.45 GHz microwave system with an input CH_4_ volume content up to 70% resulted in a hydrogen production rate of 192 g(H_2_)/h and a hydrogen energy yield of 43 g(H_2_)/kWh, both with 4.5 kW of absorbed microwave power (P_abs_). This was an important advance in the development of microwave plasma technology for efficient H_2_ production from biogas-like mixtures, but the resulting hydrogen energy yield of 43 g(H_2_)/kWh was still below the cost-effective DOE’s limit of 60 g(H_2_)/kWh. A further possible increase in the hydrogen energy yield in the 2.45 GHz system by increasing the absorbed microwave power above 4.5 kW is limited due to a thread of electrical breakdowns occurring in the typical 2.45 GHz systems at higher input microwave powers. This power limitation can be overcome by using a 915 MHz microwave system in place of a 2.45 GHz microwave plasma system. The 915 MHz microwave system safely provides more power to the microwave discharge than the 2.45 GHz one.

In this work, we present a technology based on microwave plasma for H_2_ production by steam reforming, the so-called synthetic biogas flowing through a waveguide-supplied, metal-cylinder microwave plasma source (MPS) working at 915 MHz. The used in this work synthetic biogas was a mixture of CH_4_ and CO_2_ which are the main components of a typical raw biogas.

## Experimental procedure and setup

The experimental procedure and main parts of the experimental setup used for studying the combined reforming of synthetic biogas in atmospheric-pressure microwave plasma were described in detail in our previous investigations in the dry reforming of synthetic biogas^44^. Only a general description of the experimental part of this work is provided below to assist in following up the experimental results.

**Figure 1:**
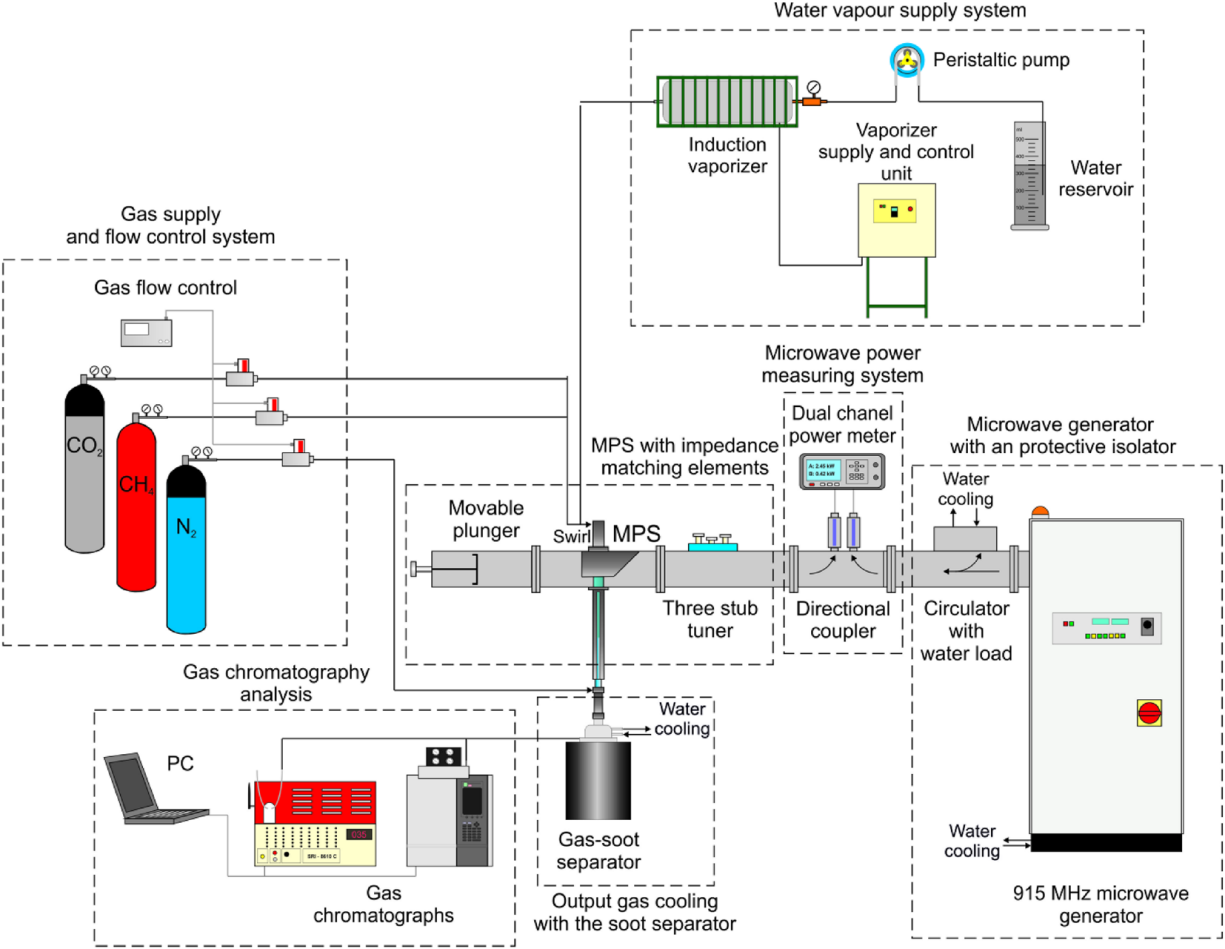
Experimental setup.

**Figure 2:**
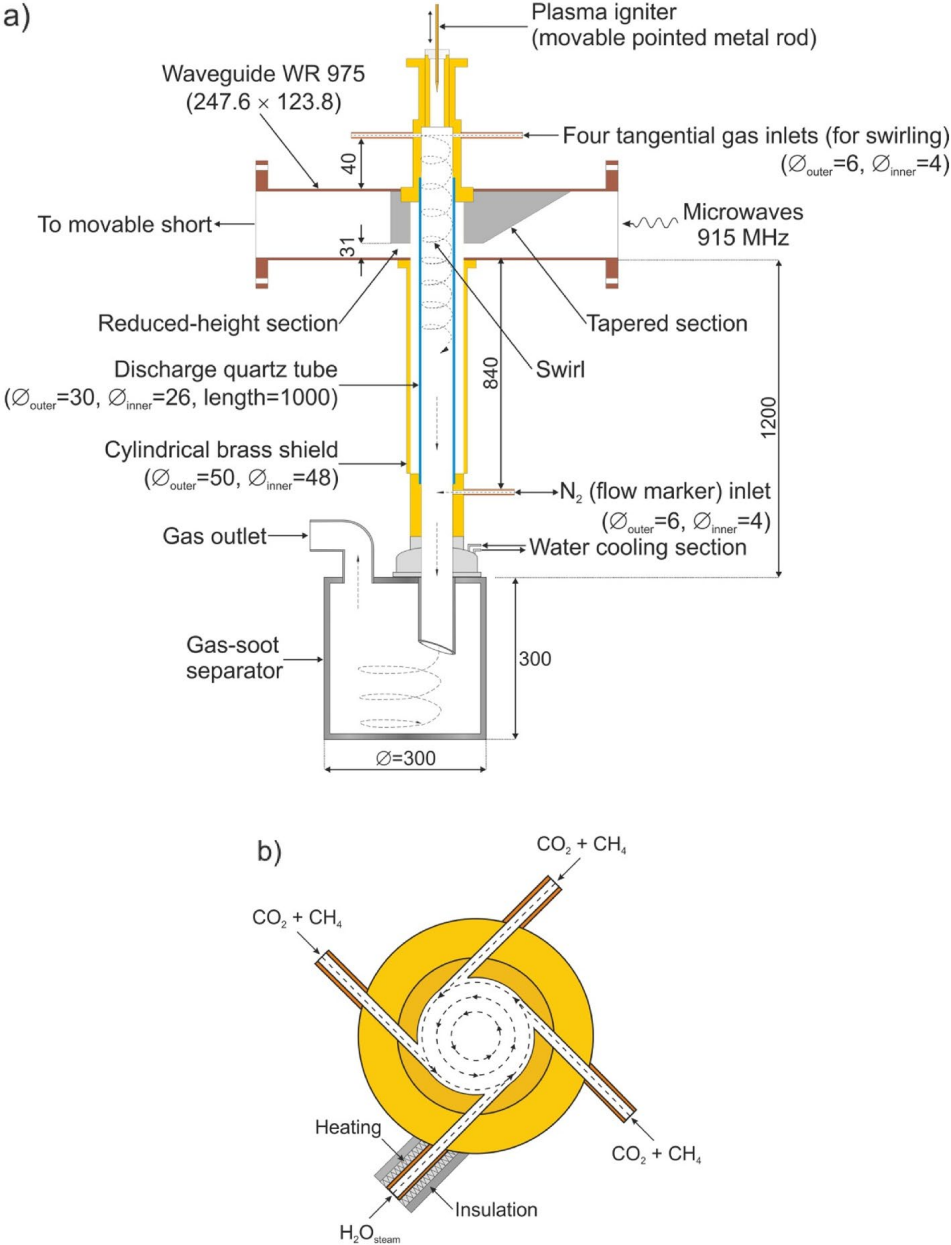
(**a**) Details of the MPS configuration (dimensions in mm), (**b**) schematic drawing of the swirl gas inlets (not in scale).

**Figure 3:**
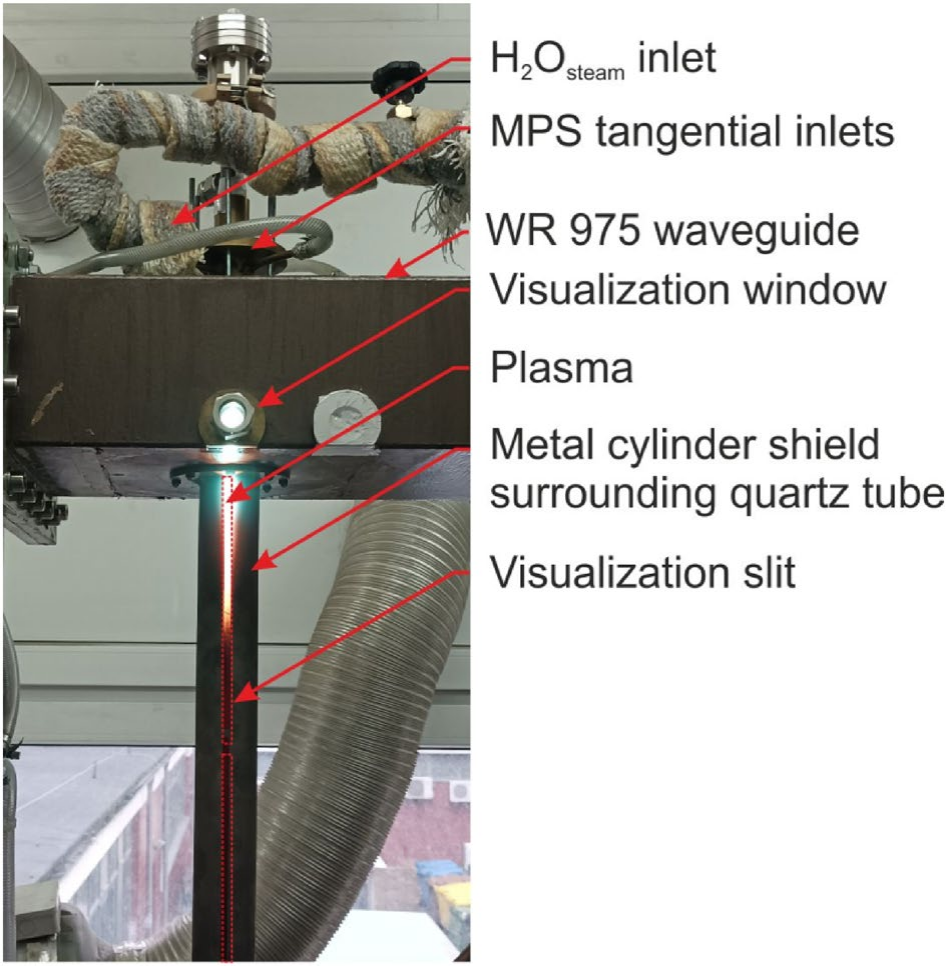
Photo showing the microwave plasma source (MPS) and the microwave plasma in the quartz tube.

The experimental setup consisted of a microwave generator (915 MHz) with a protective isolator, microwave power measuring system, impedance matching elements (three stub tuner + movable plunger), microwave plasma source (MPS), gas supply system with a flow control unit, supplemented with a water vapour supply system and a gas-soot separator unit (Fig. 1). The drawing detailing the MPS setup (with dimensions) and a schematic drawing of the swirl gas inlets are shown in Fig. 2. The MPS used in this experiment (Figs. 2 and 3) was described in detail in^45^. The experiments were performed with a synthetic gas simulated by a mixture of CH_4_ and CO_2_. Before entering the MPS, a mixture of CH_4_ and CO_2_ in various proportions was mixed with water vapour (H_2_O_steam_), together forming the working gas. The working gas in the quartz discharge tube was supplied by four tangential inlets, enabling the formation of a gas swirl flow through the tube. The mixture of CH_4_ and CO_2_ was introduced via three inlets while water vapour was introduced to one of the remaining inlets. The input CH_4_ volume content in the CH_4_:CO_2_ mixture was varied from 40 to 100%. The flow rate of the CH_4_:CO_2_ mixture through the MPS was varied from 3000 NL/h to 12,000 NL/h. In this system the H_2_O_steam_ with a temperature of 250–350 °C, was produced using an induction vaporizer, which was fed with water from a reservoir by a peristaltic pump. To avoid steam condensation the induction vaporizer outlet was placed as close as possible to the one MPS inlet which additionally was isolated. The H_2_O_steam_ consumption rate was varied from 0.5 to 3 kg/h. The microwave power absorbed by the microwave discharge changed from 4.5 to 6.5 kW, i.e. 2 kW more than in the previous dry reforming using a 2.45 GHz microwave system^44^.Under these conditions, the MPS 915 MHz worked stably at high flow rates (up to 12,000 NL/h) and high CH_4_ input volume content (up to 100%) in the CH_4_:CO_2_ mixture. The appropriate addition of H_2_O_steam_ to the CH_4_:CO_2_ mixture effectively prevented the soot formation and the extinction of the microwave discharge.

To determine the volume flow rates of the output gas components, an additional stream of N_2_ with a flow rate of 180 NL/h was introduced as a flow marker into the output gas (Figs. 1 and 2) like in^44,46,47^.

The concentrations of the output gas components were measured using a gas chromatography. For cleaning the output gas from the carbon soot formed during the methane conversion a lab-made centrifuge-type gas-soot separator was placed at the discharge quartz tube outlet. Its upper part was water cooling. Inside the gas-soot separator, the output gas was also mixed. The output gas was sampled after leaving the separator and the gas samples were collected into a Tedlar® bags to be analyzed using SRI 8610C and Shimadzu GC-2014 gas chromatographs.The presence of following components: H_2_, O_2_, N_2_, CO, CO_2_, CH_4_, C_2_H_2_, C_2_H_4_ and C_2_H_6_ in the output gas was detected and their volume flow rates were calculated.

To determine the effectiveness of the hydrogen production process the following parameters were used: hydrogen production rate, energy yield of the hydrogen production, CH_4_ conversion degree and H_2_ concentration in the output gas. The hydrogen production rate in g(H_2_)/h presents how many hydrogen is produced per hour. The energy yield of hydrogen production in g(H_2_)/kWh shows the amount of hydrogen produced consuming 1 kWh of microwave energy. The CH_4_ conversion degree is given by the ratio:

6 $$\left( {\left[ {{\text{CH}}_{{4}} } \right]_{{{\text{input}}}} - \, \left[ {{\text{CH}}_{{4}} } \right]_{{{\text{output}}}} } \right)/\left[ {{\text{CH}}_{{4}} } \right]_{{{\text{input}}}} \times {1}00,$$

where [CH_4_]_input_ is the number of CH_4_ moles at the MPS input, and [CH_4_]_output_ is the number of CH_4_ moles at the MPS output. It is the percent of the CH_4_ introduced into the microwave plasma, that was converted into non-methane products. The H_2_ concentration in the output gas determinates the percentage of H_2_ by volume is in the output gas.

In this work, the energy yield of hydrogen production and the hydrogen production rate are two basic parameters taken into account when considering the practical suitability of the presented hydrogen production method. Certainly, these are not the only factors which have to be eventually considered by the industry when deciding the implementation of the method. Other factors, like raw material extraction, manufacturing and processing, transportation, usage and retail, and waste disposal may severe influence the final implementation of the method. A method with worse energy performance, expressed by the energy yield and production rate, may be competitive and worth implementation when the others factors do not prevail. It is worth mentioning that the economic landscape is changing, and new criteria for evaluating hydrogen production may appear. Extensive research has been carried out by many governmental and private organizations all over the world to assess the hydrogen production economy at present and propose a road map for the production and distribution of hydrogen, as well as fuel cells and hydrogen systems. The most advanced assessments of the present hydrogen policy needs have been made by the U.S. Department of Energy (DOE) (e.g.,^5,48–53^, the International Energy Agency (IEA) (e.g.,^54,55^), and the European Union Commission (e.g.,^56,57^). This policy aims to identify research pathways leading to hydrogen production technologies that produce near-zero net greenhouse gas emissions and use renewable energy sources, nuclear energy, and coal (with carbon dioxide capture, utilization, and storage—CCUS). DOE evaluates the competitiveness and industrial suitability of hydrogen production methods by relating the cost of production of a kg of hydrogen to the cost of production of a gallon of gasoline (3.79 L) in the USA. At present, the cost of production of a gallon of gasoline is about US$2 (excluding delivery, storage, and tax). A gallon of gasoline is approximately equal to a kilogram of hydrogen on an energy-containing basis. Several conventional methods of mass-scale hydrogen production from fossil-originated resources (methane, natural gas and higher hydrocarbons reforming, coal gasification reforming) are currently well developed. Their hydrogen production costs are similar to that of a gasoline gallon, i.e., about US$2/kg(H_2_). However, in the future, fossil-originated technologies for hydrogen production will have to be replaced by new technologies, which will reduce the dependency on fossil resources and their impact on climate and human health and contribute to the energy supplies' reliability, stability, and security. To assess the attractiveness of new hydrogen production methods for the energy market, DOE introduced a reference cost for hydrogen production. This reference cost was set at above mentioned US$2/kg(H_2_). Also other organizations (e.g. IEA, EU Commission) evaluate that the accepted cost of hydrogen production in the Net Zero Emission Scenario in 2050 will be about US$(1–2)/kg(H_2_). This means that for new environmentally friendly technologies to be accepted in the energy market in the near future, they must meet the hydrogen production cost target of at least USD 2/kg(H_2_) unless other economic factors prevail.

The reference hydrogen production cost introduced by DOE in energy-market oriented units (US$/kg(H_2_) was modified in the scientific and technical societies to a more practical energy parameter called the energy yield expressed in units of g(H_2_)/kWh^24,25,58^, also used in this work. This modification is especially useful when electric energy is used for hydrogen production. In such cases, it is convenient to express the DOE’s reference hydrogen production cost in inverse units, that is in g(H_2_)/kWh. Assuming that the pricing of 1 kWh of electric energy is US$ 0.12 (an averaged value over the last years in the USA), the reference hydrogen production cost of US$2/kg(H_2_) corresponds to the hydrogen energy yield of 60 g(H_2_)/kWh. Thus, this value is used in this paper as the DOE’s target for the hydrogen production cost that the newly offered hydrogen production methods must meet. When the pricing of 1 kWh of electric energy is lower due to the availability of a cheap energy source (e.g., from solar or wind farms), the DOE’s target will be more advantageous for the new methods.

## Results and discussion

**Figure 4:**
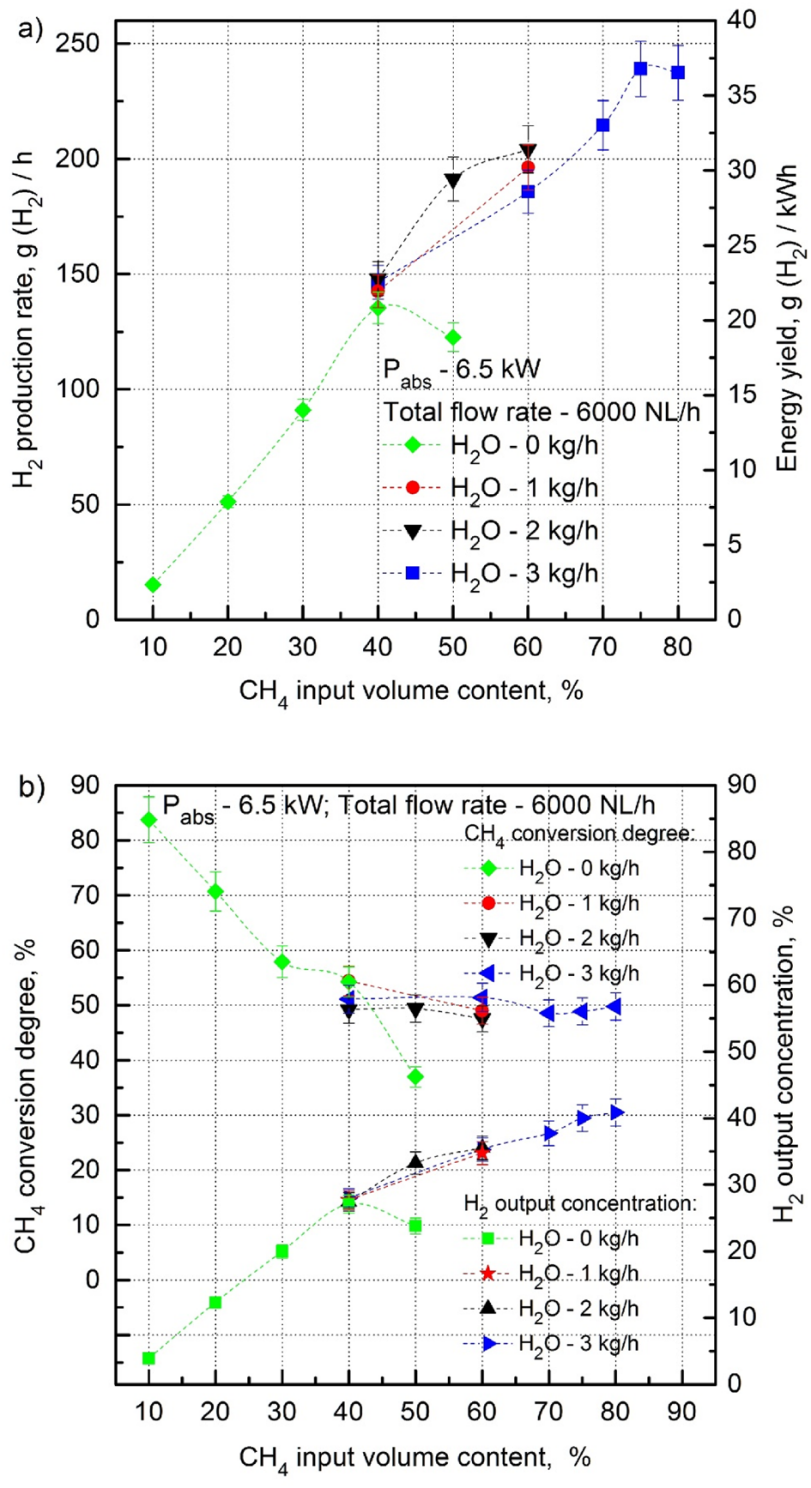
H_2_ production rate, energy yield (**a**), CH_4_ conversion degree and H_2_ output concentration (**b**) as a function of the CH_4_ input volume content in the CH_4_:CO_2_ mixture for various H_2_O_steam_ consumption rates (P_abs_—6.5 kW, total flow rate—6000 NL/h). The green measuring points (green diamond; green square) correspond to the dry reforming of the CH_4_:CO_2_ mixture [a].

As it was found in^44^, in the dry reforming process of the CH_4_:CO_2_ mixture in the 915 MHz microwave system a CH_4_ input volume content above 40% causes an increase in the amount of soot formation in the area of the microwave discharge. It causes choking of the discharge, plasma instability, and accelerated degradation of the quartz discharge tube. As a result, there is a decrease in the energy parameters of H_2_ production. This can be seen in Fig. 4a,b (the curves labeled (green diamond) and (green square), respectively), showing a clear breakdown of the growth trend of H_2_ production rate, energy yield and H_2_ output concentration with the increase of CH_4_ input volume content above 40% in the CH_4_:CO_2_ mixture. Therefore, the value of 40% of the CH_4_ input volume content determines its upper limit for the dry reforming of biogas in 915 MHz microwave plasma. The growth trend of the H_2_ production rate, energy yield and H_2_ output concentration with the increase in the CH_4_ input volume content above 40% in the CH_4_:CO_2_ mixture could be sustained by removing the cause of its breakdown which is soot production. This can be done by eliminating the generation of soot in the microwave discharge with steam (H_2_O_steam_). The addition of H_2_O_steam_ to the CH_4_:CO_2_ mixture in the microwave discharge causes oxidation of the soot produced in it, and, in consequently, quenching of the microwave discharge at high CH_4_ input volume contents ceases. This process is called steam reforming of the CH_4_:CO_2_ mixture.

**Figure 5:**
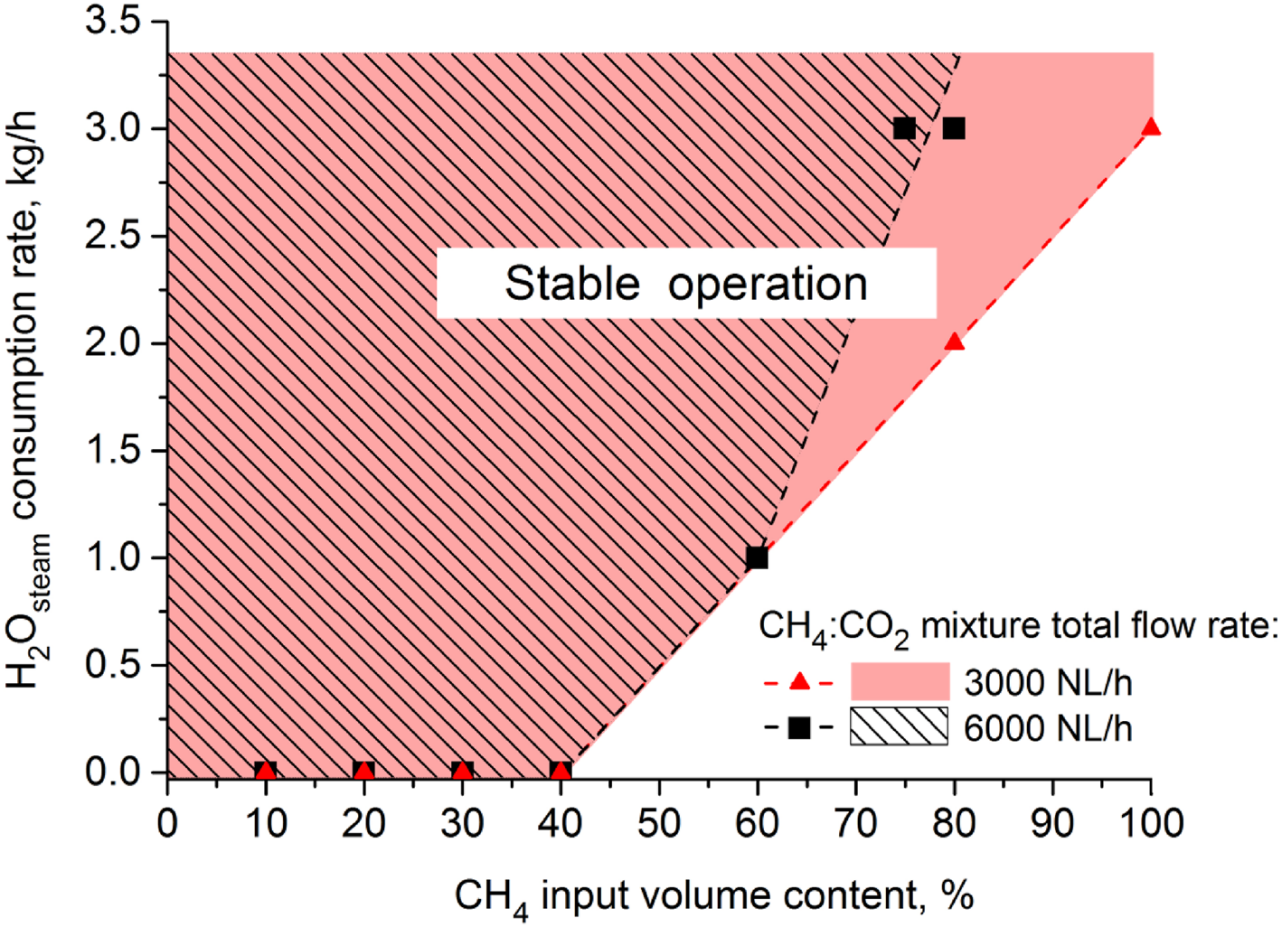
Minimal H_2_O_steam_ consumption rate (kg/h) for various CH_4_ input volume contents (%) ensuring the stable operation of the 915 MHz MPS during the steam reforming of the CH_4_:CO_2_ mixture.

Figure 5 shows the minimal amounts of H_2_O_steam_ which must be supplied to the microwave discharge in the CH_4_:CO_2_ mixture in order to eliminate the formation of soot and enable the CH_4_ input volume content to be increased above 40% during the microwave plasma reforming of the CH_4_:CO_2_ mixture. The diagram in Fig. 5 defines the areas of stable operation of the MPS during the steam reforming of the CH_4_:CO_2_ mixture for various CH_4_ input volume contents for total flow rates of CH_4_ input volume contents of 3000 NL/h and 6000 NL/h. It is worth explaining that the higher minimal H_2_O_steam_ consumption rate (kg/h) for the CH_4_ input volume content (%) of 80% at the CH_4_:CO_2_ mixture total flow rate of 6000 NL/h than that for the CH_4_:CO_2_ mixture total flow rate of 3000 NL/h is in that the absolute CH_4_ amount in NL/h for 6000 NL/h is twice that for 3000 NL/h. Thus, the former needs more H_2_O_steam_ to effectively suppress soot formation of from a higher number of CH_4_ molecules.

As it seen from Fig. 4a, at a total flow rate of the CH_4_:CO_2_ mixture of 6000 NL/h the addition of H_2_O_steam_ with a flow rate of 2 kg/h ensures stable operation of the 915 MHz MPS with a CH_4_ input volume content up to 60%. It is 20% higher than the CH_4_ input volume content limit for stable operation of the dry reforming of the CH_4_:CO_2_ mixture. The H_2_O_steam_ consumption rate of 3 kg/h ensures stable operation with a CH_4_ input volume content reaching 75%-80%. The increase in the CH_4_ input volume content from 40 to 75% almost doubled the H_2_ production rate and energy yield to 239 g(H_2_)/h and 36.8 g(H_2_)/kWh, respectively (Fig. 4a). These were the best results in terms of the H_2_ production rate and energy yield archived in this experiment (see Table 1). Figure 4a shows that above the CH_4_ volumetric content of 75%, the growth trend of the H_2_ production rate and energy yield collapses at the H_2_O_steam_ consumption rate of 3 kg/h. A possible breakdown of this trend is the insufficient amount of H_2_O_steam_ in the microwave discharges to stop soot production. However, this supposition could not be verified in this experiment due to the H_2_O vaporizer capacity being limited to 3 kg/h.

**Table 1:** Best results of the steam reforming in the presented 915 MHz MPS.

Absorbed power P_abs_ [kW]	CH_4_:CO_2_ Total flow rate [NL/h]	CH_4_ input volume content [%]	H_2_O_steam_ consumption rate [kg/h]	H_2_ production rate [g[H_2_]/h]	H_2_ energy yield [g[H_2_]/kWh]	CH_4_ conversion degree [%]	Outlet gas component concentration [volume %]
6.5	6000	75	3	**239**	**36.8**	48.9	H_2_—40.0 CO_2_—11.8 CO—14.3 CH_4_—32.0 C_2_H_2_—1.6 C_2_H_4_—0.18 C_2_H_6_—0.06
6.5	3000	100	3	234	36.1	66.1	H_2_—58.4 CO_2_—3.2 CO—14.4 CH_4_—21.1 C_2_H_2_—2.4 C_2_H_4_—0.4 C_2_H_6_—0.﻿06
6.5	3000	50	2	171.5	26.4	**74.3**	H_2_—46.78 CO_2_—13.71 CO—29.96 CH_4_—8.74 C_2_H_2_—0.56 C_2_H_4_—0.19 C_2_H_6_—0.06

Maximum values are in bold.

Figure 4b shows that in the dry reforming of the CH_4_:CO_2_ mixture, the high ability of the microwave discharge to CH_4_ conversion, expressed by the CH_4_ conversion degree (in %), weakens when the CH_4_ volume content in the CH_4_:CO_2_ mixture increases up to 40%. However, as can be easily calculated, despite the decreasing CH_4_ conversion degree, the absolute amount of CH_4_ (in NL/h) in the CH_4_:CO_2_ mixture increases resulting in an increase in the absolute amount of the converted CH_4_ (in NL/h). That is the reason that despite the decreasing CH_4_ conversion degree the H_2_ output concentration increases with the CH_4_ input volume content increase. For this reason, despite the decreasing degree of CH_4_ conversion, the output H_2_ concentration increases with the increasing CH_4_ input volume content.

Figure 4b also shows that after adding an appropriate amount of H_2_O vapour to the CH_4_:CO_2_ mixture, the CH_4_ conversion rate decreased with the increase in the CH_4_ input volume in the CH_4_:CO_2_ mixture above 40%. With a sufficient amount of H_2_O_steam_ the CH_4_ conversion degree is about 50% for the CH_4_ input volume content in the range of 40% up to 80%. Adding an appropriate amount of H_2_O_steam_ to the CH_4_:CO_2_ mixture for CH_4_ input volume contents higher than 40% causes the H_2_ output concentration to continue its increasing as the CH_4_ input volume content increases. With a CH_4_ volume content of 80%, the total flow rate of the CH_4_:CO_2_ mixture of 6000 NL/h, and an H_2_O_steam_ consumption rate of 3 kg/h, the H_2_ output concentration is about 40%.

**Figure 6:**
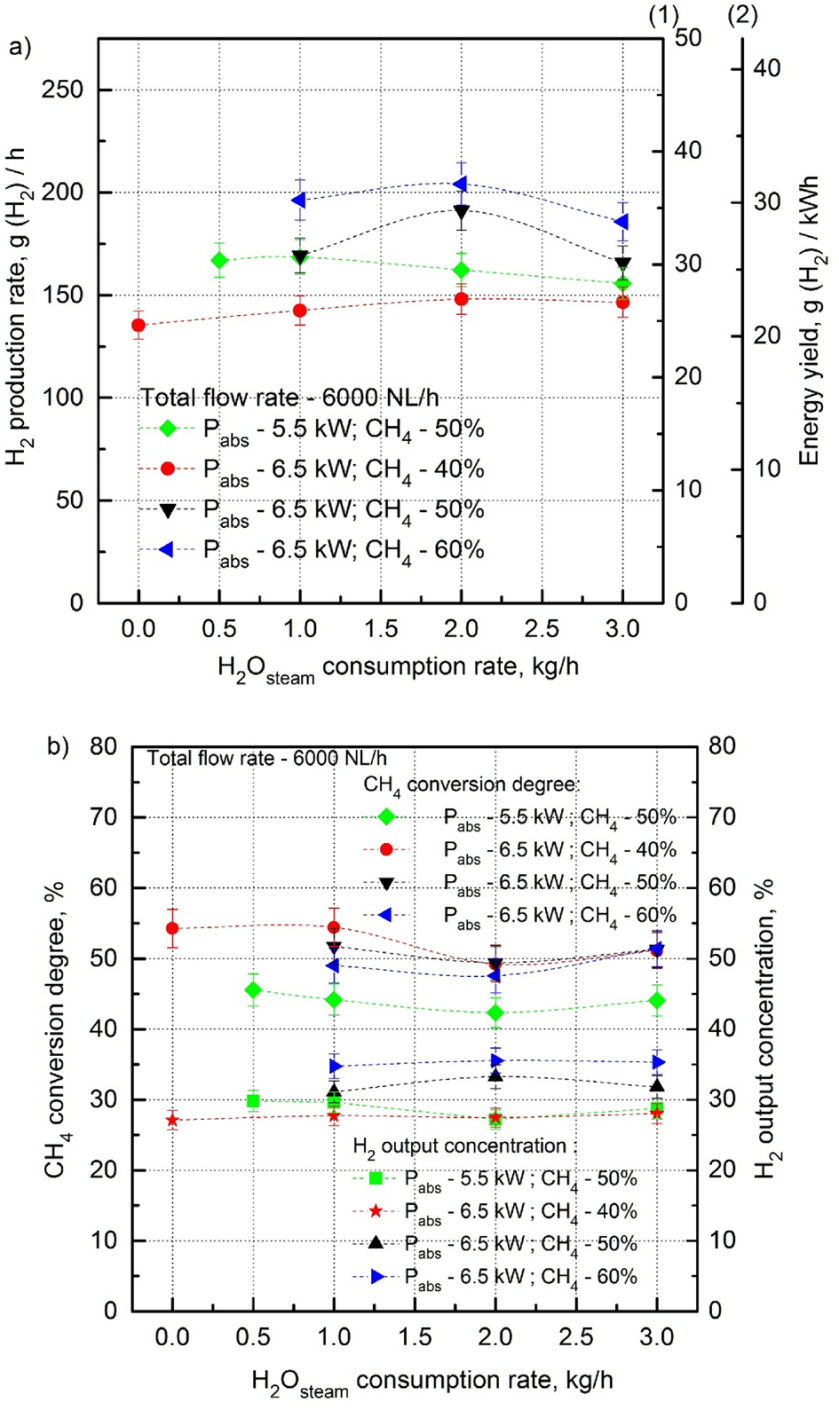
H_2_ production rate, energy yield (**a**), CH_4_ conversion degree and H_2_ output concentration (**b**) as a function of the H_2_O_steam_ consumption rate for various CH_4_ input volume contents (40–60%) and absorbed microwave powers (5.5 kW, 6.5 kW). (1) Scale for 5.5 kW, (2) scale for 6.5 kW.

It follows from the above text that the addition of H_2_O_steam_ to the microwave discharge in the CH_4_:CO_2_ mixture plays a crucial role in stabilizing the microwave discharge at higher contents of CH_4_. The minimal H_2_O_steam_ consumption rates that ensures the stable operation of the MPS during the steam reforming of the CH_4_:CO_2_ mixture at various CH_4_ input volume contents are presented in Fig. 5. As seen from Fig. 6a,b, adding H_2_O_steam_ to the microwave discharge in the CH_4_:CO_2_ mixture above the minimal amount that ensures the stable operation of the MPS for a given CH_4_ content, almost does not change the H_2_ production rate, energy yield, CH_4_ conversion degree, and H_2_ output concentration. Also, the percentages of the major components of the output gas does not change with the increasing amount of H_2_O_steam_ (Table 2 for a CH_4_ input volume content of 50% as a typical example). In other words, the surplus of amount of H_2_O_steam_ over the minimal value in the microwave discharge in the CH_4_:CO_2_ mixture does not directly improve the hydrogen production parameters. On the other hand, the H_2_O_steam_ was found to be beneficial for hydrogen production by suppressing soot production and ensuring the introduction of more CH_4_ into the microwave discharge. In consequence, more CH_4_ in the CH_4_:CO_2_ mixture results in better hydrogen production parameters (Fig. 4a,b).

**Table 2:** Percentage of the major components of the output gas for various H_2_O_steam_ consumption rate, absorbed microwave power P_abs_—5.5 kW, total flow rate—6000 NL/h and CH_4_ input volume content—50%.

CH_4_ + CO_2_ Total flow rate [NL/h]	P_abs_ [kW]	CH_4_ [%]	H_2_O_steam_ [kg/h]	H_2_ [%]	CO [%]	CO_2_ [%]	CH_4_ [%]	C_2_H_2_ [%]	C_2_H_4_ [%]	C_2_H_6_ [%]
6000	5.5	50	0.5	29.79	19.32	25.00	24.24	1.41	0.17	0.06
			1	29.60	18.72	25.95	24.47	1.04	0.16	0.06
			2	27.20	16.13	31.62	24.13	0.73	0.13	0.07
			3	28.76	16.68	27.86	25.79	0.69	0.13	0.07

Thus, the results presented in Fig. 6a,b show that introducing more H_2_O_steam_ than the minimal amount for a given CH_4_ input volume content is useless in terms of the meaningful improvement of the H_2_ production rate, and energy yield, CH_4_ conversion degree, and H_2_ output concentration.

For example, a minimal H_2_O_steam_ consumption rate of 1 kg/h is sufficient to provide the stable operation of the MPS with the best values of the H_2_ production parameters in the case of 60% of the CH_4_ input volume content at 6.5 kW of the absorbed power, and 6000 NL/h of the total flow rate of the CH_4_:CO_2_ mixture.

Within the measurement error (± 5%), the CH_4_ conversion degree and H_2_ output concentration were constans with the increasing H_2_O_steam_ consumption rate at a constant P_abs_, CH_4_ input volume content and CH_4_:CO_2_ mixture total flow rate (Fig. 6b). It suggests that the H_2_ produced in the steam reforming of the CH_4_:CO_2_ mixture comes mainly from the conversion of CH_4_, not from the conversion of H_2_O_steam_.

**Figure 7:**
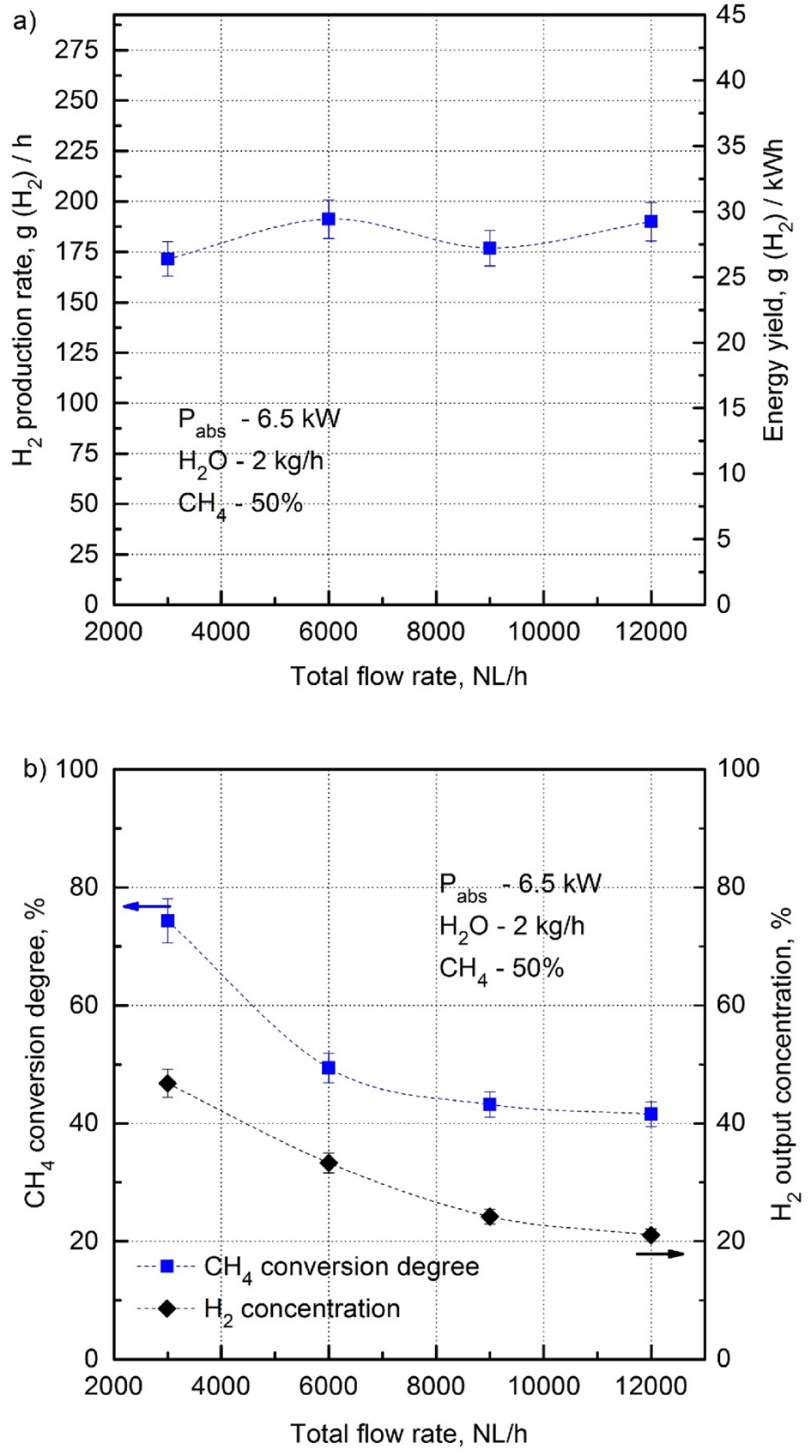
H_2_ production rate, energy yield (**a**), CH_4_ conversion degree and H_2_ output concentration (**b**) as a function of the total flow rate of the CH_4_:CO_2_ mixture (P_abs_—6.5 kW, H_2_O_steam_—2 kg/h, CH_4_—50%).

Figure 7a shows that, within the experimental error, the H_2_ production rate and energy yield remain constant with an increasing total flow rate of the CH_4_:CO_2_ mixture in the range of 3000–12,000 NL/h for the case of 6.5 kW of P_abs_, 50% of the input volume CH_4_ and 2 kg/h of the consumption rate of H_2_O_steam_. Under the same conditions, the CH_4_ conversion degree and H_2_ output concentration decrease considerably with the increasing total flow rate of the CH_4_:CO_2_ mixture (Fig. 7b). It is worth noting that although the H_2_ production rate and energy yield remain constant with an increasing total flow rate of the CH_4_:CO_2_ mixture in the range of 3000–12,000 NL/h, the percentage of unconverted CH_4_ (in other words unutilized feedstock) increases from 25% at 3000 NL/h to 57% at 12,000 NL/h. This means that the best performance of the MPS in terms of the H_2_ production rate, energy yield, CH_4_ conversion degree, and H_2_ output concentration was obtained for low CH_4_:CO_2_ mixture flow rates. This was probably due to the sufficiently long residence time of the CH_4_:CO_2_ mixture with a low flow rate in the microwave discharge, the absorbed microwave power P_abs_ of which was limited to 6.5 kW. CH_4_:CO_2_ mixtures with higher flow rates that remain for a shorter time in the microwave discharge would require a more powerful discharge to achieve a high CH_4_ conversion rate. However, if the flow rates of the CH_4_:CO_2_ mixture were too low, the cooling swirl flow inside the quartz tube could not be created and the MPS could not function properly, mainly due to overheating which could also seriously damage the MPS.

**Figure 8:**
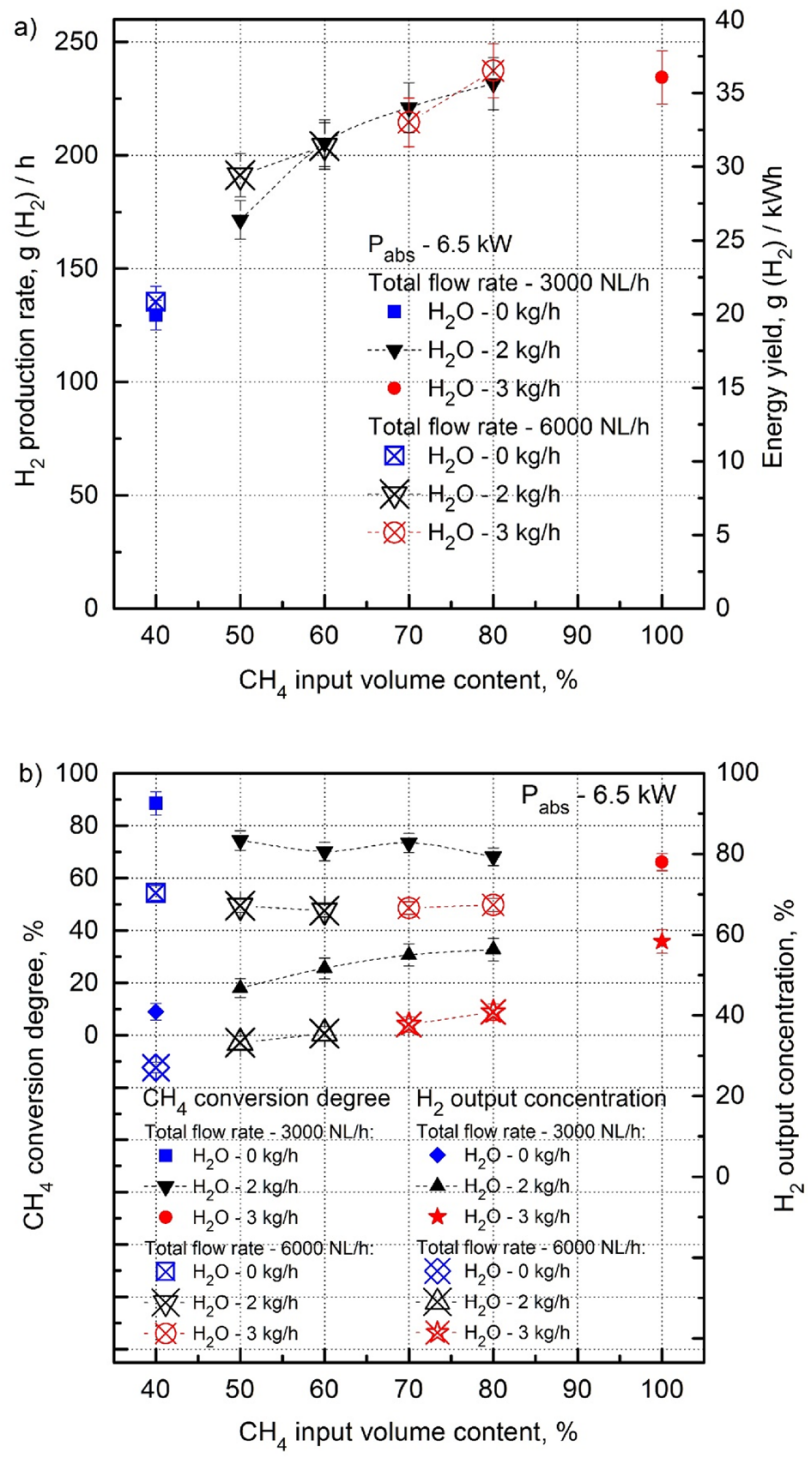
H_2_ production rate, energy yield (**a**), CH_4_ conversion degree and H_2_ output concentration (**b**) as a function of the CH_4_ input volume content (%) in the CH_4_:CO_2_ mixture for a recommended total flow rate of 3000 NL/h and, for comparison, for 6000 NL/h (P_abs_—6.5 kW). The applied H_2_O_steam_ consumption rates ensure stable operation of the MPS at a given CH_4_ input volume content.

We found out that for an absorbed microwave power of 6.5 kW the minimal total flow of the CH_4_:CO_2_ mixture ensuring safe and stable operation of the MPS without overheating and damage was 3000 NL/h. As seen from Fig. 8a, for a constant CH_4_ input volume content (in %) an increase in the total flow rate of the CH_4_:CO_2_ mixture from 3000 NL/h to 6000 NL/h did change the H_2_ production rate and energy yield. Note, however, that at a given CH_4_ input volume content, the absolute CH_4_ amount in NL/h for 3000 NL/h is half that for 6000 NL/h. This means that when working with the total flow rate of the CH_4_:CO_2_ mixture of 3000 NL/h, the same H_2_ production rate and energy yield as those with a total flow rate of 6000 NL/h are obtained with the absolute CH_4_ input amount in NL/h two times lower. This is advantageous with regard to the final H_2_ production cost that eventually also includes the cost of the CH_4_ feedstock.

As seen from Fig. 8a, in both cases (3000 NL/h and 6000 NL/h), the H_2_ production rate and energy yield increase with the increasing CH_4_ input volume content (with sufficient H_2_O_steam_ for each CH_4_ input volume content).

In the dry reforming of the CH_4_:CO_2_ mixture (at the CH_4_ input volume content of 40%)—Fig. 8b, the CH_4_ conversion degree for the total flow rate of the CH_4_:CO_2_ mixture of 3000 NL/h is higher (about 90%) than that for 6000 NL/h (about 55%). It is possibly because of the shorter residence time of the CH_4_:CO_2_ mixture in the microwave discharge at 6000 NL/h and lower specific energy input. The CH_4_ conversion degrees for 3000 NL/h and 6000 NL/h decrease as the CH_4_ input volume content (with sufficient H_2_O_steam_) increases above 40%. Then, they remain constant for the CH_4_ input volume contents higher than 50%. In this range, the CH_4_ conversion degree for the total flow rate of the CH_4_:CO_2_ mixture of 3000 NL/h is higher (about 70%) than that for 6000 NL/h (about 50%). This means that the amounts of unconverted CH_4_ are about 30% and 50% for 3000 NL/h and 6000 NL/h, respectively. The high amount (50%) of unconverted CH_4_ at 6000 NL/h shows the poor performance of the MPS at this total flow rate in terms of the utilization of the CH_4_ feedstock.

Although the CH_4_ conversion degree (in %) is approximately constant as the CH_4_ input volume content increases (from 50 to 100% with sufficient H_2_O_steam_) for the total flow rates of 3000 NL/h and 6000 NL/h, it can be calculated using Fig. 8b that in both cases the absolute amount of converted CH_4_ (in NL/h) increases with the increasing CH_4_ input volume content. This results in an increased H_2_ production rate and energy yield at higher CH_4_ input volume contents (Fig. 8a).

For both total flow rates, the H_2_ output concentration increases with the increasing CH_4_ input volume content. In the case of the total flow rate of 3000 NL/h, the H_2_ output concentration reaches about 60%, while for 6000 NL/h it is always about 16% lower. It is worth remembering that the total flow rates of the CH_4_:CO_2_ mixtures of 3000 NL/h and 6000 NL/h refer to the MPS input, while the H_2_ output concentrations are measured at the MPS output, where the output gas volume flow rates differ from those at the MPS input. For example, in the case of the CH_4_ input volume content of 60% and the H_2_O_steam_ consumption rate of 2 kg/h, the output gas volume flow rates are 4860 NL/h and 7020 NL/h for the total flow rates of the CH_4_:CO_2_ mixtures of 3000 NL/h and 6000 NL/h, respectively. The greater increase in the volume of the output gas for the CH_4_:CO_2_ mixture with 3000 NL/h can be explained by the residence time in the microwave discharge and specific energy input, twice as long and twice as that of the mixture with 6000 NL/m, respectively. It results in greater heating and an increase in the volume of the output gas due to the higher decomposition of the CH_4_:CO_2_ mixture.

Figures 7b and 8b showed the high amount of unutilized CH_4_ feedstock (from 25% at 3000 NL/h to 57% at 12,000 NL/h) during the steam reforming of the CH_4_:CO_2_ mixture in the presented MPS. This has a negative impact on the overall assessment of the performance of the MPS. However, the customary energy parameters (H_2_ production rate and energy yield) describing the performance of the MPS do not provide information on the performance of the MPS in terms of the utilization of the CH_4_ feedstock. Poor utilization of the CH_4_ feedstock causes an additional the cost of H_2_ production.

Therefore, we propose a new parameter, called the energy–CH_4_ feedstock consumption yield, to describe the MPS performance, which also takes into account a factor of the utilization of the CH_4_ feedstock. The energy–CH_4_ feedstock consumption yield shows the amount of hydrogen produced in a unit time (1 h) with the consumption of 1 kWh of microwave energy and 1 kNL/h of CH_4_ feedstock (or the ratio of the energy yield to the amount of the CH_4_ feedstock consumed in a unit time (1 h)), expressed in g(H_2_)/h/[kNL(CH_4_)/h × kWh]. For example, the energy–CH_4_ feedstock consumption yields for various total flow rates of the CH_4_:CO_2_ mixture under the conditions: P_abs_—4.5 kW, H_2_O_steam_ consumption—2 kg/m, CH_4_ input volume content—50%, Fig. 7a) are shown in Table 3. As can be seen from this Table, under the same conditions, a fourfold increase in the total flow rate of the CH_4_:CO_2_ mixture (from 3000 to 12,000 NL/h) results in an almost fourfold reduction in the energy–CH_4_ feedstock consumption yield. In other words, when increasing 4 times the total flow rate, almost 4 times less H_2_ is produced from a unit of the CH_4_ feedstock. This confirms the poor performance of the MPS at higher total flow rates of the CH_4_:CO_2_ mixtures with respect to the utilization of the CH_4_ feedstock, already reported above when discussing the results shown in Fig. 7a,b. As shown, the energy–CH_4_ feedstock consumption yield can be a useful parameter to evaluate the performance of the MPS.

**Table 3:** The energy–CH_4_ feedstock consumption yields for various total flow rates of the CH_4_:CO_2_ mixture under the conditions: P_abs_—6.5 kW, H_2_O_steam_ consumption—2 kg/h, CH_4_ input volume content—50%.

Total flow rate of the CH_4_:CO_2_ mixture [kNL(CH_4_:CO_2_)]/h	The energy–CH_4_ feedstock consumption yield, g(H_2_)/h/[kNL(CH_4_)/h × kWh]
3	17.6
6	9.8
9	6.1
12	4.9

**Figure 9:**
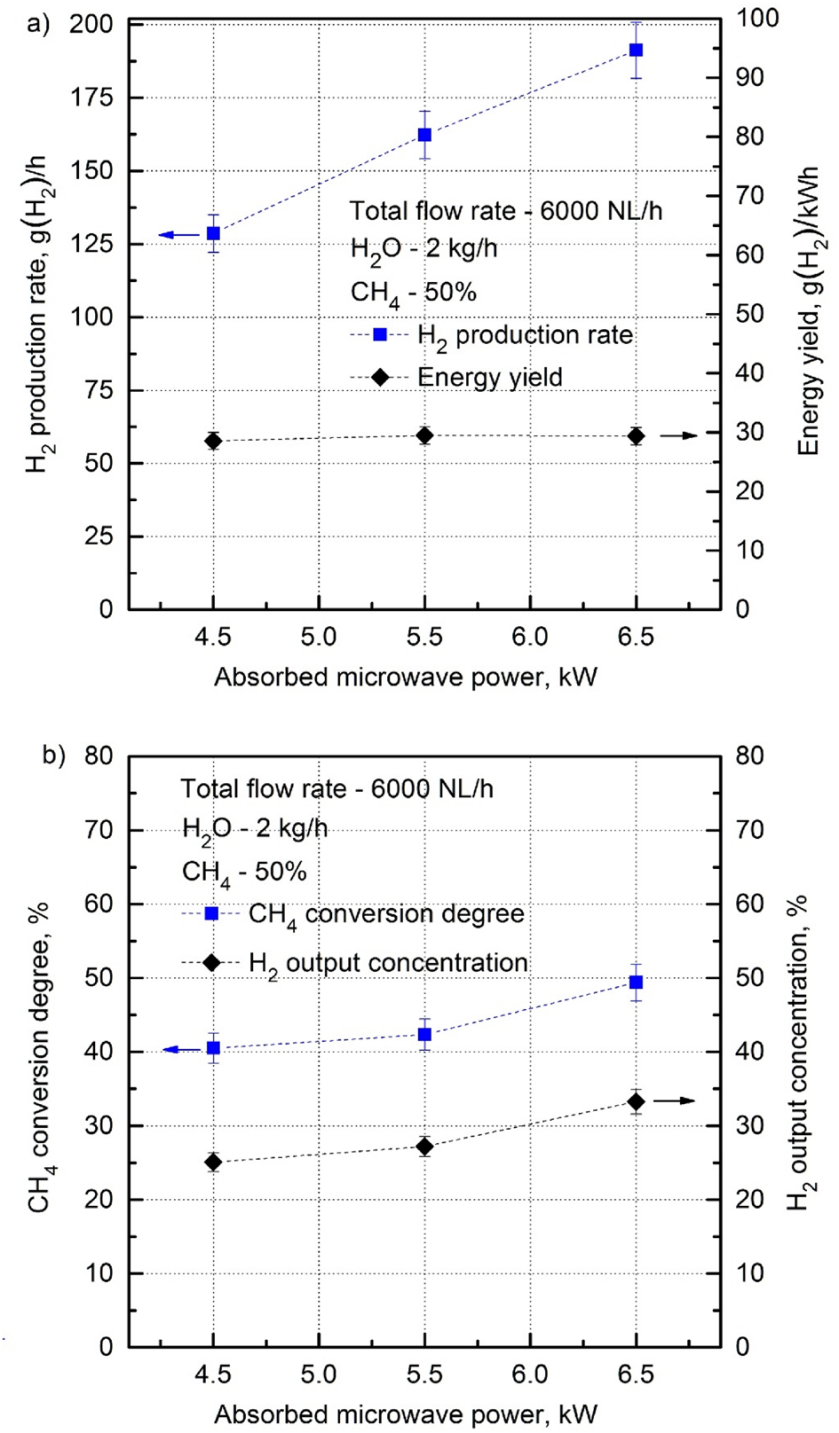
H_2_ production rate, energy yield (**a**), CH_4_ conversion degree and H_2_ output concentration (**b**) as a function of the absorbed microwave power (Total flow rate—6000 NL/h, CH_4_—50%, H_2_O_steam_—2 kg/h).

Figure 9 shows the H_2_ production parameters as a function of the absorbed microwave power P_abs_ for the total flow rate—6000 NL/h, CH_4_ input volume content—50%, and H_2_O_steam_ consumption rate—2 kg/h. As it can be seen, the H_2_ production rate, CH_4_ conversion degree and H_2_ output concentration increase with the increasing P_abs_. However, the energy yield remains constant despite the increase in P_abs_. This all means that operating with P_abs_ higher than 6.5 kW may result in higher H_2_ production (about 300 g(H_2_)/h at 10 kW, assuming a linear increase in the H_2_ production as shown in Fig. 9). However, no increase in the energy yield above 30 g(H_2_)/kWh should be expected, which is clearly lower than that assumed by the DOE.

## Summary and conclusions

This work is devoted to the development of microwave plasma-based technology aimed at the efficient production of hydrogen from biogas, which can contain up to 70% CH_4_. In the so-called upgraded biogas or bio-methane, the CH_4_ content is up to 98%. Our research to date on the suitability of microwave-based technology to produce H_2_ from the so-called synthetic biogas (a mixture of CH_4_ and CO_2_ in various proportions)^42,44^, showed that real progress in the efficiency of H_2_ production could be made if a microwave plasma system of a relatively high microwave power with a relatively high CH_4_ input volume content (at least 70%) is used. In this work, we tested the performance of a microwave plasma system operating at a frequency of 915 MHz as a tool for efficient production of H_2_ in the steam reforming of synthetic biogas. The results of these tests, widely described above can be concluded as follows.

1. By using a 915 MHz microwave plasma system we could test steam reforming of a CH_4_:CO_2_ mixture with an absorbed microwave power P_abs_ up to 6.5 kW, i.e. 2 kW more than in our previous 2.45 GHz microwave system^42^. In this work, we found that the H_2_ production rate increased as the absorbed microwave power increased to 6.5 kW.
2. The appropriate addition of H_2_O_steam_ to the synthetic biogas effectively prevented soot formation and the extinction of the microwave discharge, which previously in the dry reforming of synthetic biogas^44^ limited operation of the MPS to the CH_4_ contents of not higher than 40% (in contrary to 100% at present work).
3. We found that there is a minimal H_2_O_steam_ consumption rate for a given CH_4_ input volume content, which ensures stable operation of the MPS (no soot).
4. The experiments did not show that increasing the amount of H_2_O_steam_ rate above the minimal value for a given CH_4_ input volume content results in an increase in the H_2_ production rate, energy yield, CH_4_ conversion degree, and H_2_ output concentration. However, the H_2_O_steam_ suppresses soot production and ensures the introduction of more CH_4_ into the microwave discharge. In consequence, more CH_4_ in the CH_4_:CO_2_ mixture results in better hydrogen production parameters.
5. Within the scope of the above working conditions, the best results obtained in terms of the H_2_ production rate, energy yield, and CH_4_ conversion degree were 239 g[H_2_]/h, 36.8 g[H_2_]/kWh, and 74.3%, respectively (see Table 1). This means that the application of the steam reforming, instead of the dry reforming, resulted in a 1.5-fold increase of the H_2_ production rate and the corresponding energy yield.
6. Within the limits of the absorbed microwave power of 6.5 kW and H_2_O_steam_ consumption rate of 3 kg/h in the 915 MHz MPS used in this experiment, the best performance of the MPS in terms of the H_2_ production rate, energy yield, CH_4_ conversion degree, and H_2_ output concentration was obtained for low CH_4_:CO_2_ mixture flow rates.
7. The results showed a high amount of unutilized CH_4_ feedstock (from 25% at 3000 NL/h to 57% at 12,000 NL/h of the total flow rate) during the steam reforming of the CH_4_:CO_2_ mixture in the presented MPS. Therefore, we introduced a new parameter, called the energy–CH_4_ feedstock consumption yield, to describe the MPS performance, which also takes into account a factor of the utilization of the CH_4_ feedstock. Our calculations showed that under the same conditions, a fourfold increase in the total flow rate of the CH_4_:CO_2_ mixture (from 3000 NL/h to 12,000 NL/h) results in an almost fourfold reduction in the energy–CH_4_ feedstock consumption yield. This confirmed the poor performance of the MPS at higher total flow rates of the CH_4_:CO_2_ mixtures with respect to the utilization of the CH_4_ feedstock. The energy–CH_4_ feedstock consumption yield can be a useful parameter to evaluate the performance of an MPS.
8. Despite the progress obtained in this work (see Table 4), the energy yield of H_2_ production by the microwave plasma-based technologies (about 40 g[H_2_]/kWh) has to be improved to meet the Department of Energy’s (USA) requirement (the target of 60 g(H_2_)/kWh).
9. Better results than those obtained in this work can be expected for a higher absorbed microwave power P_abs_ (around 10 kW), higher total flow rates of the CH_4_:CO_2_ mixture with an optimal CH_4_ input volume content, and correspondingly higher H_2_O_steam_ consumption rates (higher than 3 kg/h).

**Table 4:** Progress in microwave plasma technology for hydrogen production from synthetic biogas.

Production method	Initial composition	Hydrogen production rate [g(H_2_)/h]	Energy yield [g(H_2_)/kWh]	Methane conversion degree (hydrogen selectivity) [%]	References
Conventional steam reforming of methane (catalyst)	CH_4_ H_2_O + Air	Large scale	60 Established industrial process		^5^
Metal-cylinder-based microwave plasma (2.45 GHz)	CH_4_:CO_2_ CH_4_—50%	66	19	32.5 (40.9)	^41^ 2013
Metal-cylinder-based microwave plasma (2.45 GHz)	CH_4_:CO_2_ CH_4_—50% H_2_O—2 kg/h	192	43	22 (unknown due to H_2_O presence)	^42^ 2016
Quartz-cylinder-based microwave plasma (2.45 GHz)	CH_4_:CO_2_ CH_4_—50%	112	19	96.8 (77.2)	^43^ 2017
Metal-cylinder-based microwave plasma (915 MHz)	CH_4_:CO_2_ CH_4_—40%	156	21	61.4 (63.7)	^44^ 2019
Metal-cylinder-based microwave plasma (915 MHz)	CH_4_:CO_2_ CH_4_—75% H_2_O—3 kg/h	239	36.8	48.9 (unknown due to H_2_O presence)	Present work 2022

In summarizing, taking into account the above, one could consider microwave (915 MHz) atmospheric pressure plasma-based technology for hydrogen (H_2_) production from the so-called synthetic biogas as one of the promising techniques of hydrogen production for further investigations.

## Data Availability

The datasets used and/or analysed during the current study available from the corresponding author on reasonable request.
